# Genomic and epigenomic determinants of heat stress-induced transcriptional memory in Arabidopsis

**DOI:** 10.1186/s13059-023-02970-5

**Published:** 2023-05-30

**Authors:** Christian Kappel, Thomas Friedrich, Vicky Oberkofler, Li Jiang, Tim Crawford, Michael Lenhard, Isabel Bäurle

**Affiliations:** Institute for Biochemistry and Biology, University of Potsdam, 14476 Potsdam, Germany

**Keywords:** Transcriptional memory, Priming, Heat stress, HSFA2, HSFA3, Arabidopsis thaliana, ChIP-seq, Histone H3K4 trimethylation

## Abstract

**Background:**

Transcriptional regulation is a key aspect of environmental stress responses. Heat stress induces transcriptional memory, i.e., sustained induction or enhanced re-induction of transcription, that allows plants to respond more efficiently to a recurrent HS. In light of more frequent temperature extremes due to climate change, improving heat tolerance in crop plants is an important breeding goal. However, not all heat stress-inducible genes show transcriptional memory, and it is unclear what distinguishes memory from non-memory genes. To address this issue and understand the genome and epigenome architecture of transcriptional memory after heat stress, we identify the global target genes of two key memory heat shock transcription factors, HSFA2 and HSFA3, using time course ChIP-seq.

**Results:**

HSFA2 and HSFA3 show near identical binding patterns. In vitro and in vivo binding strength is highly correlated, indicating the importance of DNA sequence elements. In particular, genes with transcriptional memory are strongly enriched for a tripartite heat shock element, and are hallmarked by several features: low expression levels in the absence of heat stress, accessible chromatin environment, and heat stress-induced enrichment of H3K4 trimethylation. These results are confirmed by an orthogonal transcriptomic data set using both de novo clustering and an established definition of memory genes.

**Conclusions:**

Our findings provide an integrated view of HSF-dependent transcriptional memory and shed light on its sequence and chromatin determinants, enabling the prediction and engineering of genes with transcriptional memory behavior.

**Supplementary Information:**

The online version contains supplementary material available at 10.1186/s13059-023-02970-5.

## Background

Plants can be primed by an exposure to a moderate stress to better withstand a recurrent stress event [1–3]. This has been shown for biotic and abiotic stressors, including heat stress (HS) [3–5]. Boosting the capability of crop plants for priming against critical stresses may increase stress tolerance and secure yield in times of changing climates [6]. At the molecular level, priming against HS is associated with two types of transcriptional memory: (1) the sustained induction of HS-induced gene expression that lasts several days longer than the priming HS (type I) and (2) enhanced transcriptional re-induction after a recurrent HS (type II) [7, 8].

The molecular basis underlying transcriptional memory is largely unknown. In a broader context, transcriptional memory is found in plants also after drought, salt, and pathogen-related stress exposure [4, 9–13]. It also occurs in a developmental context, such as in epigenetic silencing of gene expression, which can be mediated by endogenous or environmental cues [14–16]. Transcriptional memory was also described in response to changes in the available carbon source in yeast [17–19] and in the interferon-γ response in mammalian cells [20]. A shared feature of these phenomena is the correlation with certain histone modifications, namely histone H3 lysine 4 di-/trimethylation in the case of an activating or potentially activating memory [9, 11, 12, 18, 19]. However, what distinguishes a promoter that displays transcriptional memory after a recurrent signal from one that responds identically to the signal every time it occurs remains an unresolved question.

A model case for transcriptional memory is HS memory in *Arabidopsis thaliana* [5]. Genes that show type I and type II transcriptional memories after HS have been catalogued, and several factors have been identified that are necessary for the transcriptional memory [7, 8, 21–23]. However, the underlying molecular mechanisms are presently not clear, as is the question what distinguishes memory genes from HS-inducible non-memory genes. Type I memory requires the FORGETTER1 (FGT1) protein, the orthologue of *Drosophila* strawberry notch [23]. In the nucleus, FGT1 interacts with chromatin remodeling proteins of the SWI/SNF and ISWI classes to maintain low nucleosome occupancy throughout the memory phase, thus promoting active transcription [23]. In addition, two heat shock transcription factor (HSF) transcription factors, HSFA2 and HSFA3, are required for sustained induction (type I) memory after HS [7, 22, 24].

The HSF family is highly conserved across kingdoms and universally promotes transcriptional responses to high temperatures, but also tumorigenesis and aging responses [25, 26]. In *A. thaliana*, the HSF family has 21 members, of which eight have been implicated in the HS response [27–29]. The three HSFA1 isoforms HSFA1A, HSFA1B, and HSFA1D are required for the early HS responses [27, 28, 30–33]. They are constitutively expressed and maintained in an inactive state by binding to chaperone proteins. Upon HS, the chaperones are recruited to other proteins, thereby activating the HSFA1 isoforms. They then induce transcription of chaperone genes until they are inactivated by vacant chaperones (chaperone titration model). HSFs in all organisms are known to form homo- and heteromeric complexes, typically trimers or hexamers [25, 34, 35]. Because of the multitude of HSF isoforms in *A. thaliana*, it is unclear which isoforms complex with each other. HSF proteins bind to extended repeats of the sequence nGAAn, which may alternate in their orientation and are called heat shock elements (HSEs), either as homomeric or heteromeric trimers [36, 37]. Whether individual HSFs have differing sequence preferences is not clear.

HSFA2 and HSFA3 (jointly termed memory HSFs) are specifically required for HS memory but not for the initial acquisition of thermotolerance. They are strongly induced by HS, with *HSFA2* being directly induced by HSFA1s, and *HSFA3* in part by DREB2 family proteins, which are themselves induced by HSFA1, resulting in slower activation kinetics [31, 32, 38, 39]. Curiously, single mutants in *hsfa2* and *hsfa3* have strong memory phenotypes, only slightly less severe than the double mutant [22]. Both HSFs are required for the correct expression of genes such as *APX2* and *HSP22* during HS memory and bind to their promoters, where they mediate sustained hyper-methylation of histone H3K4 [7, 22]. HSFA2/HSFA3 interact with each other and with additional HSFs. These findings support the model that HSFA2/HSFA8isplay full activity only if both proteins are present in the same complex. It remains unclear which part of the proteins specifies them as memory HSFs. Notably, HSFA2 and HSFA3 form complexes with other HSFs in the absence of the respective other memory HSF [22]. While both proteins function in a highly related manner, there is the possibility that their targets are only partially overlapping. Previous candidate approaches have identified and confirmed a small number of direct targets of HSFA2 and HSFA3 [7, 22]. These include not only memory genes but also HS-inducible non-memory genes such as *HSP101* [40].

To determine the genome-wide set of HSFA2/HSFA3 target genes and to identify distinguishing features between memory and non-memory genes, we performed time-course ChIP-seq of HSFA2 and HSFA3. We analyzed the expression and chromatin organization of the targets genes and compared them to in vitro binding data. We find that HSFA2 and HSFA3 jointly bind their targets. Binding is largely determined by DNA sequence, with a strong enrichment of a tripartite HSE-like motif. Besides, memory genes are characterized by low expression pre-HS, the absence of histone modifications, and a strong enrichment of H3K4me3 after HS. Thus, our findings shed light on the molecular determinants of transcriptional memory in response to environmental stress.

## Results

### Binding profiles of HSFA2 and HSFA3 are highly similar, but they differ on different sets of genes

**Fig. 1:**
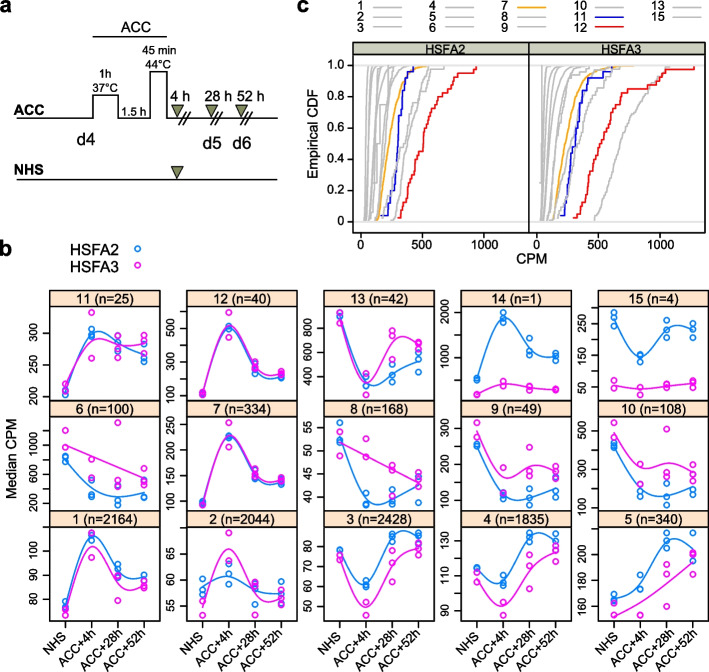
HSFA2 and HSFA3 targets during HS memory fall into 15 clusters and show differential binding strength and dynamics. **a** Treatment schematic for ChIP-seq and RNA-seq experiments. Four-day-old seedlings were exposed to a two-step acclimation treatment (ACC) of the indicated temperature and duration. Sampling was at the indicated time points after the end of ACC (arrowheads) or at the corresponding time point for no-HS (NHS) samples. **b** Median ChIP-seq signal of HSFA2 and HSFA3 in 15 cclusters at the indicated time points. CPM, counts per million; *n*, number of peaks in each cluster. **c** Memory cclusters show high binding strength at 4 h after ACC. Empirical cumulative distribution function of HSFA2- and HSFA3-bound peaks at 4 h after ACC. C7, c11, and c12 are highlighted in color. Binding of c12 is significantly higher than for all other clusters (HSFA2) and than for all other clusters except c6 (HSFA3), respectively (KS-test, *p* < 0.05, Additional File 2: Supplementary Data 1). Cluster c14 is not included as it contains only one peak

To determine the direct targets of memory HSFs and their binding dynamics we performed ChIP-seq analysis of unstressed seedlings and seedlings at different times after a priming HS (termed ACC, with recovery for 4 h, 28 h, or 52 h, Fig. 1a), expressing Flag-HSFA2 or Flag-HSFA3 from the respective endogenous promoters. Both transgenic lines show full complementation of the *hsfa2* and *hsfa3* phenotypes (Additional File 1: Fig. S1) [22]. We selected the time points 4 h, 28 h, and 52 h after the end of a HS in line with previous analyses. Since both HSFs are strongly induced after HS [7, 22, 24], no-HS (NHS) samples contain only small amounts of tagged protein and were expected to yield low chromatin binding. For each time point, three biological replicates were performed.

**Fig. 2:**
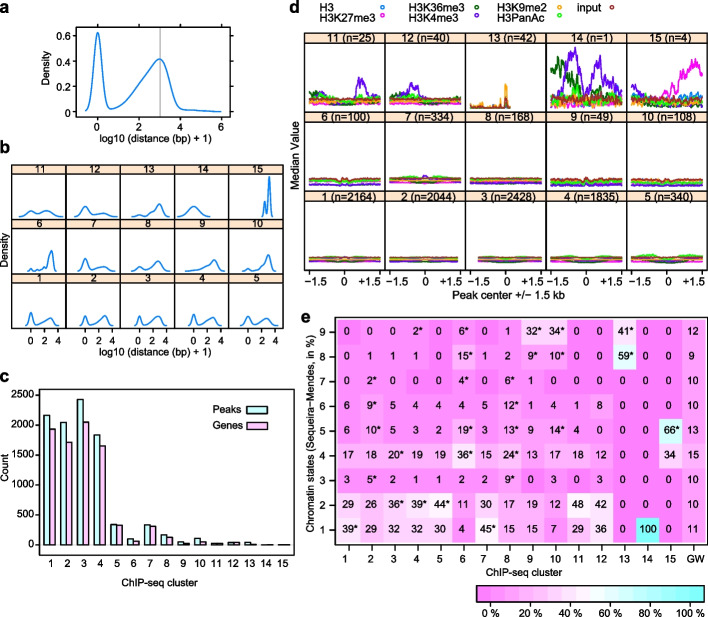
Characterization of binding sites of HSFA2/HSFA3 according to their distance from associated genes, chromatin profiles, and chromatin states. **a** Density plot illustrating global distance of peaks from the nearest transcriptional start site (TSS). Gray line indicates distance of 2000 bp. **b** Density plot illustrating global distance of peaks from the nearest transcriptional start site (TSS) in ChIP-seq clusters. **c** Number of peaks and number of associated genes in cclusters. **d** Enrichment of histone modifications in seedlings grown under control (NHS) conditions in a window of 3 kb around the peak centers. Ccluster c11, c12, and c14 are enriched for H3K4me3 at the flanks of the peaks, cluster c13 is enriched for H3K9me2, and cluster c15 is enriched for H3K27me3. Histone modification data were reanalyzed from [42]. **e** ChIP-seq peaks are enriched in different chromatin states, with clusters c7, c11, c12, and c14 being enriched in chromatin states 1 and/or 2. The proportion of peaks in cclusters that are associated with the indicated chromatin states is depicted. *p < 0.05 Fisher’s exact test. Chromatin states are taken from [43]

The initial peak calling resulted in several thousand peaks that were present in at least one of the samples. Peak width was adjusted to 200 bp, centering on the summit. Compared to the 74 consolidated HSF binding sites in yeast [41], this is a comparatively high number, suggesting that not all of these peaks may be functionally relevant. However, to minimize bias due to filtering with hard thresholds, we used hierarchical clustering to sort the peaks into 15 different ChIP-seq clusters (cclusters) depending on their binding profile over the time course (Fig. 1b, c). Overall, HSFA2 and HSFA3 displayed remarkably similar binding patterns. In terms of median signal strength over all time points, six clusters have a very low signal (c1-5, c8), three clusters (c6, c13, c14) have an exceedingly high signal, and the remaining six clusters (c7, c9-12, c15) have an intermediate signal strength. Because *HSFA2* and *HSFA3* expression is HS-inducible, we expect true targets to show low (or no) binding in NHS conditions. Clusters c6, c8, c9, c10, c13, and c15 showed the highest signal at NHS, suggesting they do not represent true HSF targets. In contrast, c1, c2, c5, c7, c11, c12, and c14 showed an increase in signal strength after HS, conforming to our expectations. Thus, biologically relevant targets may be concentrated in seven clusters, together comprising 4948 peaks. Interestingly, c12 showed the highest binding signal at 4 h compared to all other clusters (for HSFA2) and all other clusters except c6 (for HSFA3), respectively (KS-test, *p* < 0.05, Additional File 2: Supplementary Data 1). We next determined the distance of the peaks to the closest transcriptional start site (Fig. 2a–c). Overall, we found a biphasic distribution, with a large fraction of peaks very close to a TSS and another large fraction several hundred to more than 1000 bp away (Fig. 2a). Notably, clusters c7 and c12 contained a much higher proportion of peaks at the TSS than distant peaks; conversely, clusters c6, c8, c9, c10, and c13 were dominated by distal peaks (Fig. 2b). In some cases, one gene was associated with more than one peak, thus there are slightly fewer genes than peaks in the clusters (Fig. 2c).

### Chromatin states in clusters

To start to address whether different clusters are enriched in certain histone modifications before HS, we analyzed the average profile of 5 frequently profiled histone modifications (H3PanAc (acetylation), H3K4me3, H3K9me2, H3K27me3, H3K36me3) [42]. We observed enrichment of H3K4me3 in clusters c11, c12, c14, of H3K9me2 in c13, of H3K36me3 in c14, and of H3K27me3 in c15; all other clusters did not show any enrichment in any modification (Fig. 2d). Notably, the enrichment in H3K4me3 and H3K27me3 was not observed in the center of the peaks, but at a distance of several hundred base pairs, indicating that there is no specific marking of the HSF binding site, but rather that the modifications occur in the bodies of associated genes.

Chromatin modifications including DNA methylation and histone modifications tend to occur together in certain combinations that determine the overall activity state and accessibility of the chromatin [43, 44]. A recent study in *A. thaliana* defined nine chromatin states [43]. The promoters of active genes and genes amenable to rapid activation tend to be in the “open” chromatin states 1 and 2, which are enriched in H2A.Z, H3K4me2, and H3K4me3. We investigated whether the clusters differ in their chromatin states. Cclusters c1-5, c7, c11, c12, and c14 are enriched in chromatin states 1 and 2 (Fig. 2e). Conversely, c9, c10, and c13 are enriched in chromatin states 8 and 9, representing heterochromatic chromatin organization (enriched in DNA methylation and H3K9me2), and c6 and c15 in states 4 and 5, representing silenced chromatin enriched in the polycomb mark H3K27me3. Notably, all clusters with a HS-induced increase in HSF binding (clusters c1, c2, c5, c7, c11, c12, c14) are enriched in open chromatin that is typical for promoter regions (states 1 and 2), suggesting that these clusters are enriched in biologically relevant target genes (Figs. 1b, 2e). Together with the overall binding strength, this suggests that clusters c5, c7, c11, c12, and c14 represent biologically relevant targets of HSFA2/HSFA3.

### Three clusters contain highly HS-inducible genes with reduced expression in *hsf* mutants

**Fig. 3:**
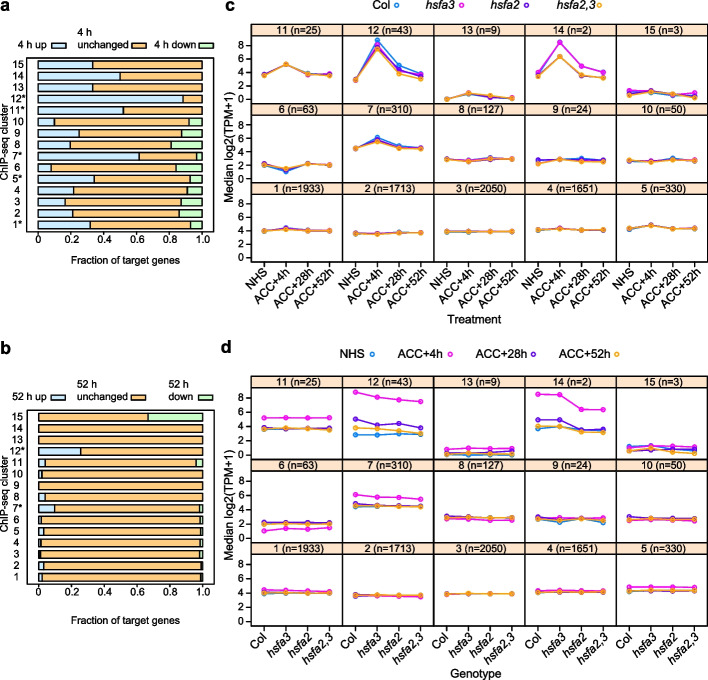
HSFA2 and HSFA3 target genes display HS-induced sustained gene expression that depends on memory HSFs. **a** Fraction of genes in ChIP-seq clusters that show differential expression in wild type at 4 h after ACC. Clusters c1, c5, c7, c11, and c12 are significantly enriched in genes that are upregulated at this time point (*p < 0.01, Fisher’s exact test, Additional File 2: Supplementary Data 1). **b** Fraction of targets genes in ChIP-seq clusters that show differential expression in wild type at 52 h after ACC. Clusters c7 and c12 are significantly enriched in genes that are upregulated at this time point (*p < 0.01, Fisher’s exact test, Additional File 2: Supplementary Data 1). **c**,** d** Median expression of genes in ChIP-seq clusters at the indicated time points and genotypes. Gene expression in clusters c7, c12, and c14 is dependent on HSFA2/HSFA3. HS-induced expression in clusters c12, c14, and to a lesser extent c7 shows sustained induction in Col-0 wild type. *n*, number of genes per cluster

We next asked whether the increased binding of memory HSFs to genes in clusters c1, c2, c5, c7, c11, c12, and c14 is reflected in induced gene expression after HS. To this end, we integrated the ChIP-seq data with transcript profiling data of Col-0, *hsfa2*, *hsfa3*, and *hsfa2 hsfa3* double mutants at the corresponding time points (NHS, 4 h, 28 h, 52 h after ACC, Fig. 1a) [22]. Genes that are upregulated at 4 h after ACC are significantly enriched in cclusters c1, c5, c7, c11, and c12 (*p* < 0.01, Fisher’s exact test, Fig. 3a, Additional File 2: Supplementary Data 1). Genes that are upregulated at 52 h after ACC are enriched in c7 and c12 (*p* < 0.01, Fisher’s exact test, Fig. 3b, Additional File 2: Supplementary Data 1). The median expression of all of these clusters peaked at 4 h (Fig. 3c, d). Sustained induction at 28 and 52 h was evident in c12 and c14 and weakly in c7 and c11 (Fig. 3d). The induction after ACC depended on functional HSFA2 and HSFA3 in c7, c12, and c14 (Fig. 3c, d). While the genes in clusters c1 and c5 were overall slightly induced by ACC, this was not dependent on memory HSFs (Fig. 3c). This is consistent with the finding that these genes did not show sustained induction. Thus, c12 and c14 (and to a lesser extent c7) are enriched for genes with HSFA2/HSFA3-dependent and overall sustained induction of gene expression during the memory phase. While c12 is associated with 43 genes, c14 has only one peak that is associated with *HSFA2* and its neighbor gene. C12 contains many *HSP* genes and several previously identified and confirmed targets such as *HSP22.0*, *HSP101*, and *MIPS2* (Additional File 1: Fig. S2, Additional File 3: Supplementary Data 2, [7, 8, 22]). While sustained induction of c7 is less clear than for c12 and c14 based on median gene expression, c7 contains several previously characterized HS-memory genes, including *APX2*, *HSA32*, and *HSP18.2* [7, 22]. Thus, clusters c7, c12, and c14 are enriched in biologically relevant targets of HSFA2 and HSFA3. We experimentally validated the binding of HSFA2/HSFA3 to five putative target genes from c7 and c12 by ChIP-qPCR (Additional File 1: Fig. S3) and found that the results were fully consistent with previous reports [7, 22] and the time course ChIP-seq analysis reported here.

### Binding of memory HSFs is largely determined by sequence

**Fig. 4:**
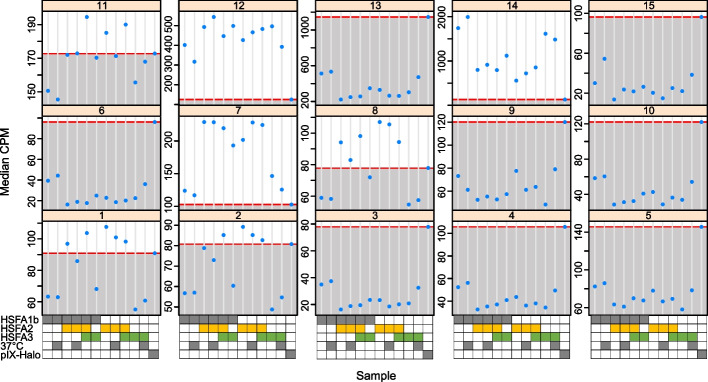
The binding intensity of memory HSFs is largely determined by sequence, with modulations from composition of heteromeric complexes. The indicated combinations of HSFA2, HSFA3, and HSFA1b were allowed to bind to genomic DNA at the indicated temperatures (37 °C or 22 °C) and purified and analyzed by DAP-seq. Signal intensities at the peaks from the ChIP-seq experiment were determined and averaged for each ccluster. The red line indicates the signal intensity in the pIX-Halo control sample, which only expressed the Halo-tag. Specific binding intensities over background are highest in clusters c7, c12, and c14

To investigate to what extent DNA sequence determines target selection and binding strength of HSFA2/HSFA3, we performed in vitro binding assays (DAP-seq) [45] for HSFA2, HSFA3, HSFA1b, and combinations thereof. During the binding reaction, samples were incubated at either 25 °C or 37 °C to analyze the effect of temperature on binding. In contrast to ChIP-seq, DAP-seq assesses transcription-factor binding to ‘naked’ DNA in the absence of nucleosomes, but with in vivo levels of DNA methylation [45]. The previously published transcription factor-wide DAP-seq experiment [45] did not include HSFA2 or HSFA3. We mapped the DAP-seq reads to the *A. thaliana* genome and determined normalized read counts for the ChIP-seq clusters defined above. The highest cluster-wide binding intensity was found for clusters c7, c11, c12, and c14 (Fig. 4), in strong agreement with the ChIP-seq results. For c11, binding above the pIX-Halo background was only seen for some of the samples, all of which contained HSFA2. C8 and to some extent c1 and c2 showed binding above the pIX-Halo negative control at least for some combinations. Interestingly, where both temperatures were tested binding was not generally stimulated by incubation at 37 °C. With the exception of HSFA3, individual HSFs bound DNA similarly as their heteromeric combinations, suggesting a minor role of complex formation. Interestingly, for clusters c7 and c12, binding by HSFA2 and/or HSFA3 appeared stronger than binding by HSFA1b. Thus, our DAP-seq results indicate that DNA sequence (and not chromatin structure) is a major factor determining the binding affinities of memory HSFs and confirms c7, c11, c12, and c14 as highly relevant target gene clusters.

### Binding motifs in the clusters

**Fig. 5:**
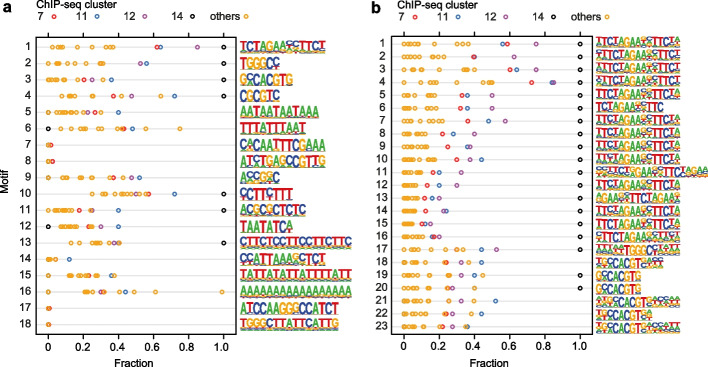
Sequence motifs underlying ChIP-seq peaks. **a** De novo motif analysis was performed with HOMER on clusters c7, c11, c12, and c14. The fraction of peaks with the indicated motif is shown for each ccluster. Motifs are sorted according to increasing *p*-value (cf. Additional File 4: Supplementary Data 3). The most highly enriched motif contains the core motif AGAAnnTCTT consisting of two conserved HSE elements [46]. **b** Transcription factor binding motif analysis was performed with HOMER on clusters c7, c11, c12, and c14. The fraction of peaks with the indicated motif is shown for each ccluster. Motifs are sorted according to increasing *p*-value (cf. Additional File 4: Supplementary Data 3). The first 16 motifs contain variations of HSE elements

The observed binding profiles suggest that the promoter DNA sequence is an important determinant of binding intensity. Thus, we asked whether particular sequence motifs are enriched under the ChIP-seq peaks in the clusters. A de novo motif analysis using HOMER2 identified 18 motifs as enriched under the peaks in c7, c11, c12, and c14 compared to the rest of the genome. Of these, only the HSE-like motif (motif 1 in Fig. 5a, Additional File 4: Supplementary Data 3) was present under more than 50% of the peaks. In addition, a TCP targeting motif (TGGGCC, motif 2 in Fig. 5a) was found under more than 50% of the peaks in clusters c11, c12, and c14. By contrast, the other 16 motifs did not reach such a high enrichment in the memory clusters. In a complementary approach, we compared the sequences under the peaks to known transcription-factor binding motifs. This identified 22 motifs, most of which were variations on a tripartite HSE (Fig. 5b, Additional File 4: Supplementary Data 3). In particular, variants of tripartite HSE motif TTCtaGAAnnTTCt (motifs 1–16) were strongly enriched in the main memory cclusters c12 (90%) and c14 (100%), as well as cclusters c7 (67%) and c11 (84%), but at much lower frequencies in the remaining cclusters. Among the known motifs identified in this analysis, there was again the TCP targeting motif (motif 17 in Fig. 5b), as well as the G-box motif CACGTG (motifs 19, 20); however, especially the latter motif was present in less than half of the peaks in the main memory clusters. Thus, while no single motif fully discriminated between peaks in memory vs. non-memory clusters, strong binding of HSFA2 and HSFA3 to the tripartite HSE motif TTCtaGAAnnTTCt contributes to the sustained expression of HS memory genes.

### Transcriptome clustering provides an orthogonal analysis

**Fig. 6:**
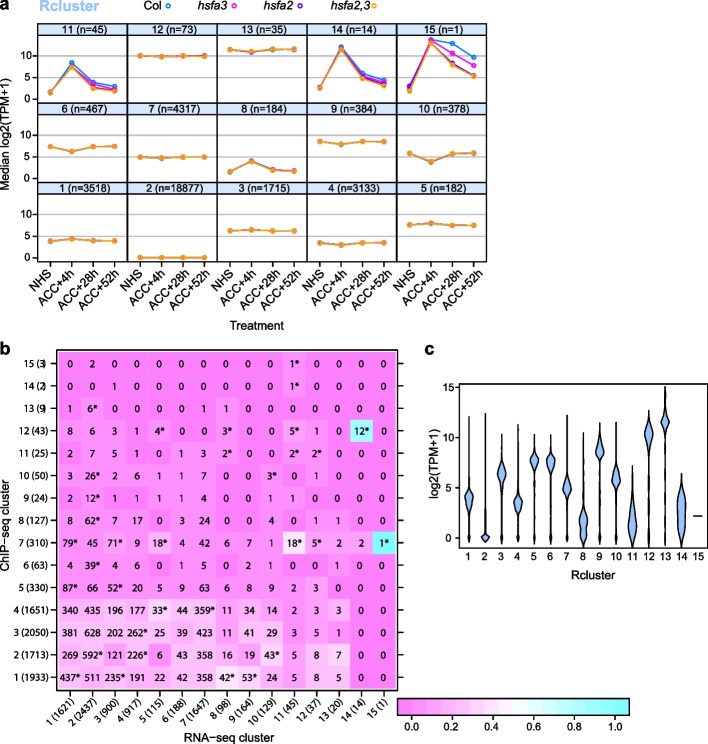
Transcriptomics cluster analysis, baseline expression and overlay with ChIP-seq clusters. **a** Median RNA-seq expression signal of Col-0, *hsfa2*, *hsfa3*, and *hsfa2 hsfa3* double mutant in 15 rclusters at the indicated time points. tpm, transcripts per million; *n*, number of genes per cluster. **b** Heat map illustrating the overlap of genes in rclusters and cclusters. Colors indicate the fraction of genes in rclusters found in ccluster. Numbers in brackets after cluster number indicate the total number of elements in the respective cluster. *p < 0.05 Fisher’s exact test. **c** Violin plot representing the basal expression level (at NHS condition) of each rcluster. Rclusters r2, r8, r11, and r14 show low basal expression

HSFA2 and HSFA3 are transcriptional activators that regulate HS memory by maintaining sustained gene expression [7, 22]. Hence, expression of direct target genes is expected to be sustained after ACC and less sustained and possibly less induced in *hsfa2* and *hsfa3* mutants. In an orthogonal approach to the ChIP-seq, we performed hierarchical clustering on a transcriptome dataset, covering the same treatments and time points, and all mutant combinations (Fig. 1a). Of the 15 RNA-seq clusters (in the following referred to as rclusters, Additional File 5: Supplementary Data 4), three were enriched in strongly HS-inducible genes with sustained expression; all three were dependent on both HSFA2 and HSFA3 (rclusters r11, r14, r15, Fig. 6a). R1, r5, and r8 contained genes that were somewhat induced at 4 h after ACC, and r6, r10, and r13 contained genes that were somewhat repressed at 4 h after ACC, but recovered thereafter (Fig. 6a). Thus, rclusters r11, r14, and r15 are likely enriched in direct target genes of HSFA2 and HSFA3. Indeed, they contain previously characterized targets (*HSA32*, *APX2*, *MIPS2*, *HSP101* in r11; *HSP22.0* in r14; *HSP18.2* in r15).

We next determined the overlap of genes in the cclusters and rclusters (Fig. 6b). Rcluster r15 only contains *HSP18.2*, and this is in cluster c7. The 14 genes from r14 were all in either c12 (12 genes including *HSP22.0*) or c7 (2 genes). Of the 45 genes from r11, more than half (24 genes) were in c7 (18), c12 (5), or c14 (1), respectively. Thus, there is a striking and significant overlap between the cclusters with the strongest HS-induced binding (c7, c12, c14) and the rclusters with the strongest, most sustained, and HSF-dependent HS induction (r11, r14, r15) (Fig. 6b, p < 0.05, Fisher’s exact test). R11, r14, and r15 showed the highest fold-induction at 4 h after HS and the highest sustained induction at 28 h and 52 h. Notably, they also had low baseline expression (Fig. 6c). Besides, several HS-induced genes with lesser fold-induction at 4 h and no or little sustained induction were present in r1, r5, and r8. However, their expression did not depend on HSFA2/HSFA3 at the cluster level. Nevertheless, a substantial fraction of genes in these rclusters were still bound by memory HSFs, mostly in c1, c2, and c7. While memory HSFs may contribute to their transcriptional activation after ACC, they are not required for the HS-induction of these genes probably because other HSFs can substitute. Thus, HS-induced sustained and HSFA2/HSFA3-dependent expression is highly correlated with strong binding of HSFA2/HSFA3, low baseline expression (NHS), and an overall high fold-induction. In summary, by using orthogonal approaches, we identified a highly overlapping set of targets that are enriched in the biologically meaningful features HSFA2/3-dependent expression and HS-induced HSFA2/3 binding.

### Overlap with established definitions of memory genes

**Fig. 7:**
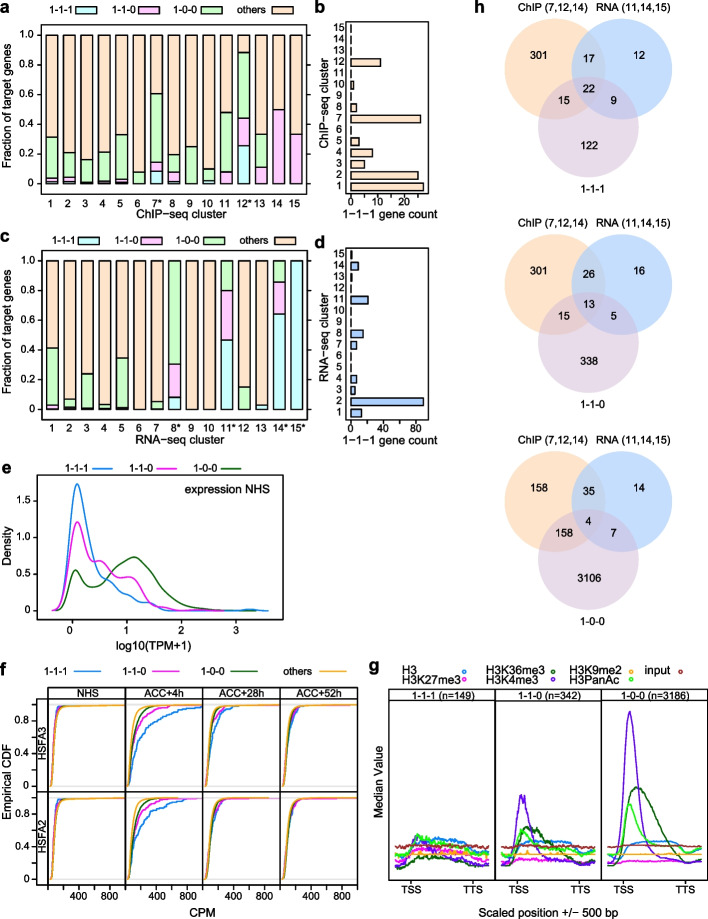
Integration of –omics data sets with a previous definition of HS memory genes. As described previously, 1–1-1 genes are upregulated at 4 h, 28 h, and 52 h after ACC, 1–1-0 genes are upregulated at 4 h and 28 h after ACC, and 1–0-0 genes are upregulated only at 4 h [22]. **a**, **b** 1–1-1 genes are enriched in cclusters c7 and c12 (*p < 0.01, Fisher’s exact test, Additional File 2: Supplementary Data 1). The fraction of memory genes across cclusters (**a**) and the number of 1–1-1 genes per cluster (**b**) is indicated. **c**, **d** 1–1-1 genes are enriched in rclusters r8, r11, r14, and r15 (*p < 0.01, Fisher’s exact test, Additional File 2: Supplementary Data 1). Fraction of memory genes across rclusters (**c**) and the number of 1–1-1 genes per rcluster (**d**) is indicated. **e** 1–1-1 genes have a low basal expression level. Density plot representing the basal expression of 1–1-1, 1–1-0 and 1–0-0 genes. **f** 1–1-1 genes show increased binding (CPM) of HSFA2 and HSFA3 after ACC. Empirical cumulative distribution function of HSFA2- and HSFA3-binding at the indicated time points after ACC. The distribution of values for 1–1-1 genes was significantly different to that of the other three groups for HSFA2 at 4 h and for HSFA3 at 4 h and 28 h (KS-test, *p* < 0.05). **g** 1–1-1 genes are not enriched in any histone modification. Enrichment of histone modifications at memory genes of different categories. Gene bodies ± 500 bp are shown; gene models were scaled between transcriptional start site (TSS) and transcriptional termination site (TTS). *n*, number of genes per group. Histone modification ChIP-seq data were reanalyzed from [42]. **h** Venn diagrams illustrate the overlap between HSFA2/HSFA3 target genes according to binding (c7, c12, c14) and expression (r11, r14, 15) and 1–1-1, 1–1-0, 1–0-0 genes, respectively

Previous studies defined type I HS memory genes as genes that show upregulation at 4 h after ACC and sustained induction until 52 h into the recovery phase (1–1-1 genes) [21, 22]. In contrast to the above cluster analysis, this was based on differential gene expression with a hard threshold. We were curious to determine the overlap between the clusters identified in this study and the previously defined 1–1-1 memory genes, early heat inducible genes (1–0-0), and genes that are upregulated at 4 h and 28 h, but not at 52 h (1–1-0), respectively. 1–1-1 genes were significantly enriched in cclusters c7 and c12 (*p* < 0.01, Fisher’s exact test, Fig. 7a, b, Additional File 2: Supplementary Data 1) and in rclusters r8, r11, r14, and r15 (*p* < 0.01, Fisher’s exact test, Fig. 7c, d, Additional File 2: Supplementary Data 1). While r8 contained some 1–1-1 genes, it was dominated by 1–0-0 genes. Thus, 1–1-1 genes strongly align with the ChIP-seq and RNA-seq clusters that are enriched in functional HSFA2/HSFA3 targets and 22 of the 168 1–1-1 genes were shared with c7, c12, c14 and r11, 14, 15 (Fig. 7h, Additional File 6: Supplementary Data 5). The promoters of these 22 genes showed further enrichment of HSEs, compared to the rest of the 1–1-1 genes (Additional File 6: Supplementary Data 5).

Like r11, r14, and r15, 1–1-1 genes show low baseline expression when compared to 1–0-0 and 1–1-0 genes (Fig. 7e). Clusters c7 and c12 showed the highest proportion of HS-induced genes at 4 h (Fig. 7a), and rclusters r8, r11, r14, and r15 contained exclusively HS-responsive genes (Fig. 7c). Overall, binding of HSFA2 and HSFA3 at 4 h was stronger in 1–1-1 genes compared to the other groups and the genome-wide average; for HSFA3, this was true also at 28 h (Fig. 7f, KS-test, *p* < 0.05, Additional File 2: Supplementary Data 1). Under no-HS conditions, we did not find a notable enrichment of any histone modification among 1–1-1 genes (Fig. 7g). Thus, memory gene sets defined previously by differential expression or expression cluster analysis (this work) show a strong overlap with memory gene sets based on HSFA2/HSFA3 binding.

### H3K4me3 enrichment after HS is a prominent feature of memory genes

**Fig. 8:**
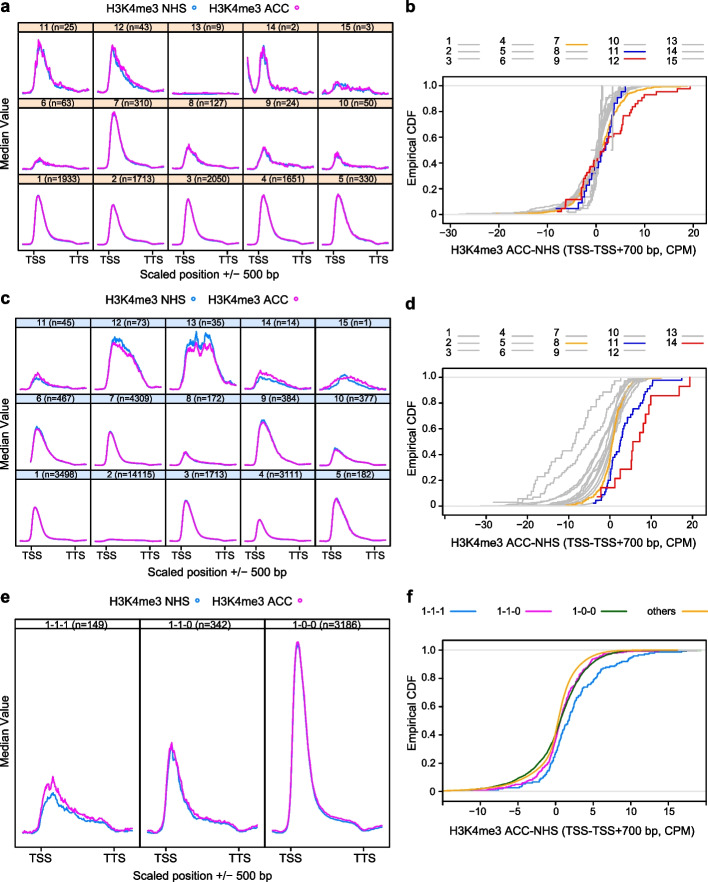
H3K4me3 is globally enriched at memory genes after 3 days of recovery. **a**, **b** Cclusters c7, c11, and c12 show increased H3K4me3 3 days after ACC. Enrichment of histone H3K4me3 at NHS and 3 days after ACC treatment in ccluster-associated genes (**a**). Gene bodies ± 500 bp are shown; gene models were scaled between transcriptional start site (TSS) and transcriptional termination site (TTS). Empirical cumulative distribution function of differential H3K4me3 accumulation (TSS to TSS + 700 bp) after ACC with c7, c11, and c12 highlighted in color (**b**). The distribution of values for memory clusters c7 and c12 was significantly different from that of the five large non-memory clusters c1 to c5 (KS-test, *p* < 0.05). **c**, **d** Rclusters r11, r14, and r15 show increased H3K4me3 3 days after ACC. Enrichment of histone H3K4me3 at NHS and 3 days after ACC treatment in rcluster-associated genes (**c**). Empirical cumulative distribution function of differential H3K4me3 accumulation (TSS to TSS + 700 bp) after ACC with r8, r11, and r14 highlighted in color (**d**). The distribution of values for rclusters r11 and r14 was significantly different from that of all other clusters (KS-test, *p* < 0.05). **e**, **f** 1–1-1 genes show increased H3K4me3 3 days after ACC. Enrichment of histone H3K4me3 at NHS and 3 days after ACC treatment in memory-associated genes (**e**). Empirical cumulative distribution function of differential H3K4me3 accumulation after ACC (TSS to TSS + 700 bp) (**f**). The distribution of values for 1–1-1 genes was significantly different from that of all other groups (KS-test, *p* < 0.05). *n*, number of genes per category

HS-induced transcriptional memory is correlated with the enrichment of H3K4me3 at known memory loci [7, 8, 22]. We thus asked whether this accumulation is observed globally at HSFA2/3-dependent memory genes. To this end, we analyzed the H3K4me3 enrichment after a HS treatment and 72 h of recovery from a published data set [47]. Overall, H3K4me3 enrichment peaked shortly after the TSS, irrespective of the condition (Fig. 8). C7 and c12 (and to some extent c11 and c14) showed hyper-accumulation of H3K4me3 at 72 h of recovery (Fig. 8a). The differential signal of H3K4me3 at 72 h of recovery in the TSS + 0.7 kb interval was significantly higher in c7 and c12 compared to the five large non-memory clusters c1 to c5 (Fig. 8b, Additional File 2: Supplementary Data 1, KS-test, *p* < 0.05). Similarly, transcriptome clusters r11, r14, and r15 showed pronounced HS-induced H3K4me3 hyper-methylation with values for r11 and r14 higher than for all other clusters (Fig. 8c, d, Additional File 2: Supplementary Data 1, KS-test, *p* < 0.05). This was not observed in any of the other clusters including r8, which contains HS-induced genes that lack sustained induction and are independent of HSFA2/3. Thus, these analyses provide strong genome-wide support for H3K4me3 hyper-methylation as a hallmark of transcriptional memory after HS. To further test the overall relevance of H3K4me3 hyper-methylation, we calculated the average modification levels at 1–1-1 genes. 1–1-1 genes display hyper-methylation of H3K4me3 after HS and long recovery with significantly higher values than for the other groups (Fig. 8e, f, Additional File 2: Supplementary Data 1, KS-test, *p* < 0.05). Taken together, sustained H3K4me3 enrichment after HS is a global hallmark of HS memory genes.

**Fig. 9:**
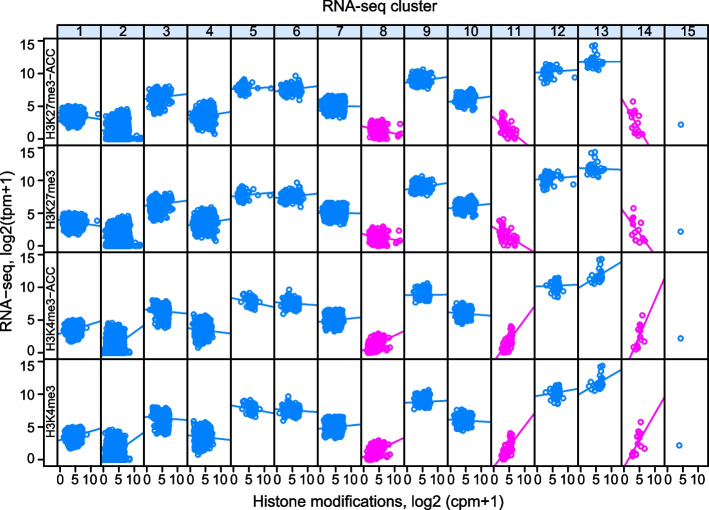
Correlation of H3K4me3 and H3K27me3 to NHS expression in RNA-seq clusters. H3K4me3 is strongly positively correlated with expression in rclusters r11 and r14, while H3K27me3 is strongly negatively correlated. For all other clusters this correlation and anti-correlation is less strong. Histone modification enrichment at NHS or ACC + 76 h (from [47]) is plotted against baseline expression in Col-0

This in turn suggests that expression of memory genes may be more sensitive to histone modification levels than that of non-memory genes. To address this, we asked whether HS memory genes in clusters r11 and r14 have a different relationship between histone modifications and expression levels in the non-treated state than non-memory genes. Regressing NHS expression levels on histone modification across the RNA-seq clusters indicated that r11 and r14 indeed show the steepest positive regression line for H3K4me3 and conversely the steepest negative regression line for H3K27me3 (Fig. 9). This does not appear to be simply a result of the generally low expression of HS memory genes before HS, as genes in r4 with a similar baseline expression level do not show any dependence of gene-expression levels on either of the two histone marks.

## Discussion

HSFA2 and HSFA3 have been identified as key memory HSFs that play a crucial role during HS-mediated sustained induction and transcriptional memory. In this study, we identified a genome-wide set of memory genes that are direct targets of HSFA2/A3 and whose expression is dependent on both factors. These memory targets of HSFA2/A3 are characterized by a combination of six features. (i) They show strong HS-dependent binding of HSFA2 and HSFA3 close to their TSS mediated by a tripartite HSE motif (Figs. 1, 2, 4 and 5). (ii) They are lowly expressed before HS (Figs. 3, 6, and 7), and (iii) the sites bound by HSFA2/A3 have few distinctive histone modifications before HS (Figs. 2 and 7). (iv) Despite the low expression before HS, the promoters of these genes are in an open chromatin state (Fig. 2), consistent with high accessibility to transcriptional regulators. (v) A distinctive feature of their chromatin after HS and long recovery (3 days) is an accumulation of histone H3K4me3 (Fig. 8). (vi) Expression of the genes appears to be particularly sensitive to the level of H3K4me3 in their promoters (Fig. 9).

Notably, binding of HSFA2/HSFA3 is not exclusive to memory genes. Many genes are bound whose expression does not depend on HSFA2/HSFA3. Thus, there are several tiers of targets. Core targets are situated in clusters c12 and c14. They show particularly strong binding, high induction after HS, sustained induction, and dependency on memory HSFs. The second tier of targets is found in c7 and c11; they do not fulfill all criteria, but many genes in these clusters nevertheless constitute functional targets based on previous analysis [8, 22]. Second tier targets are presumably also bound by other HSFs. Thus, it will be interesting to compare our findings to previously reported genome-wide targets of HSFA1a and b isoforms [48, 49].

The target genes of HSFA2 and HSFA3 overlap strongly. This confirms at a genomic scale previous findings that both genes act in a heteromeric complex and are functionally closely related [22]. Either memory HSF can form complexes with other HSFs in the absence of the other memory HSF [22]. However, the lack of specific targets argues that this does not occur in wild type plants and is consistent with the strong phenotypes of the single mutants that are almost as severe as those of the double mutant.

The enrichment of HSE sequences in all cclusters with strong heat-induction of expression (c7, c11, c12, c14) confirms the binding of HSFA2 and HSFA3 to HSEs. In particular, virtually all of the peaks in the core memory clusters c12 and c14 contained a variant of the tripartite motif TTCtaGAAnnTTCt, indicating that the HSFA2/3 memory complex has a particularly high affinity for this sequence. The tripartite nature of the motif is fully consistent with binding by a HSF trimer, with each HSF contacting a single TTC triplet [50, 51]. Notably, we were unable to identify a strictly memory-specific HSE motif, raising the hypothesis that other features are involved in target gene selection. The argument for an important role of the DNA sequence (and possibly cytosine methylation) derives from our DAP-seq results that assess binding of proteins to ‘naked’ genomic DNA without histones, but with cytosine methylation patterns intact [45]. In these, binding strength of HSFA2 and HSFA3, alone or in complex with each other or with HSFA1b, correlates closely with the strength of HS-induced binding in ChIP-seq, with peaks in c7 and c11 bound more weakly than those in c12, and the single peak in c14 showing the strongest binding in both experiments. In our DAP-seq, temperature only has a minor effect on overall binding strength, as we did not find a general stimulation of binding by incubation at 37 °C. This is not unexpected as HSFA2/HSFA3 are known to bind and activate target gene expression during the recovery phase at normal growth temperatures [7, 22]. Interestingly, this was also true for HSFA1b, which is one of the HSFs that mediate the early transcriptional induction at acute HS [32, 33]. Thus, neither protein appears to undergo major conformational changes by the temperature shift that would enable or abrogate its DNA binding ability. The high degree of overlap of binding patterns between the DAP-seq and ChIP-seq data also rules out major influences of additional unidentified factors for the binding of HSFA2 and HSFA3.

Other features that characterize HSFA2/A3 binding sites in core targets relate to their chromatin environment; strongly bound sites in vivo are found close to the TSS in the promoters of genes with low expression under control temperatures, yet with an open, transcription-competent chromatin state and no enrichment of any particular histone modification around the binding sites. The impact of histone modifications on transcription-factor binding has been well established [52], and the observed relationship between strong binding and an open chromatin conformation is fully consistent with previous findings. The low expression of memory genes before HS is particularly evident from the comparison with HS-induced non-memory genes (1–1-1 vs. 1–0-0 genes in Fig. 7e). We do not think that this is merely a statistical artifact, with sustained expression being easier to detect for genes with a very low baseline expression, as this baseline expression for r14 that contains the highest fraction of 1–1-1 memory genes is actually higher than for r8 that includes mostly HS-induced non-memory genes (Fig. 6c).

Strong binding of HSFA2/A3 to core memory loci is associated with an accumulation of H3K4me3 after HS and 72 h of recovery, as seen most strongly for r11 and r14 that overlap largely and fully with c7 and c12, respectively. This association had been established at the level of individual loci before [7, 22]. In contrast, the result presented here is based on independent genome-wide data sets generated in different laboratories and therefore provides the strongest evidence yet for a robust genome-wide association of sustained expression and H3K4me3 accumulation after HS at memory loci. It provides the first genome-scale evidence for a role of H3K4me3 in transcriptional memory, transcending numerous reports on this function at the single-gene level [9, 11, 12, 18, 20]. Based on results from individual loci, the HSFA2/A3-containing trimer appears to recruit histone methyltransferases to these loci [7], causing the H3K4me3 accumulation. Testing whether this is true genome-wide and identifying the recruited enzymes will be important future steps.

The HSFA2/A3-dependent memory genes in r11 and r14 do not only accumulate H3K4me3 after HS, but as a group their transcriptional output also appears to be particularly sensitive to the level of this histone modification, judging from the strong positive correlation between baseline expression and H3K4me3 levels across the two clusters. What determines the relationship between histone modification levels and transcriptional activity of a locus is not well understood. Also, while suggestive, the above correlations across the clusters do not show how the transcriptional activity of an individual locus responds to modulation of histone modification levels. Nevertheless, these findings lend urgency to the task of determining whether H3K4me3 hyper-accumulation causes sustained expression of memory genes or merely accompanies it.

## Conclusions

In summary, our findings shed light on the molecular determinants of transcriptional memory in response to environmental stress in plants. In particular, we provide an integrated view of HSF-dependent transcriptional memory and its sequence and chromatin determinants. These findings will contribute to the prediction and engineering of genes with transcriptional memory, thus providing novel targets for improving stress tolerance in crops. Second, we provide a global framework for how environmentally mediated transcriptional activation by HSFs is extended beyond the duration of the external cue by transcription factor-dependent histone modifications, thus mediating phenotypic plasticity. More generally, HSFs are tightly linked to the control of proliferative growth in tumorigenesis and proteotoxic stress defense in aging. Beyond HSFs, our work sheds light on the fundamental question of how chromatin stores environmental information for extended time periods.

## Methods

### Plant materials, growth conditions and HS treatments

All *A. thaliana* lines used in this study are in the Col-0 background. *pHSFA3::3xFlag-HSFA3 hsfa3* was described previously [22]. Plants were grown on GM medium (1% [w/v] glucose) under a 16 h/8 h light/dark cycle at 23/21 °C. For HS treatments for ChIP-seq, 4-day-old seedlings were exposed to a two-step acclimation (ACC) protocol consisting of 37 °C for 1 h, 23 °C for 90 min, and 44 °C for 45 min. Samples for ChIP-seq were harvested 4 h, 28 h, or 52 h after the end of the ACC treatment. Non-HS (NHS) samples were harvested at the same time as ACC + 4 h samples. For maintenance of acquired thermotolerance assay, 4-day-old seedlings were exposed to the ACC protocol described above and 3 days later to a HS at 44 °C of 70–110 min as indicated. Seedlings were imaged 14 days after ACC.

### Construction of *3xFlag-HSFA2* line

To obtain *pHSFA2::3xFlag-HSFA2 hsfa2*, a 560 bp promoter fragment flanked by *Asc*I and *Age*I restriction sites (primers 2624/2625) was amplified, as was a fragment flanked by *Age*I and *Not*I restriction sites comprising the *HSFA2* gene including an N-terminal 3xFlag-tag and downstream region (primers 3268/3269). Both fragments were subcloned into pJET1.2 (Thermo Fisher). After sequencing, the fragments were introduced into a pGreenII binary vector with Norflurazone resistance. The construct was introduced into *Agrobacterium tumefaciens GV3101* and *hsfa2* plants were transformed using the floral dip method. qRT-PCR was performed as described [22]. Oligonucleotide sequences are available in Additional File 1: Table S1.

### Chromatin immunoprecipitation

Cross-linking and immunoprecipitation of samples was performed as described previously [22, 53]. Briefly, *3xFlag-HSFA2* and *3xFlag-HSFA3* seedlings were harvested at the indicated time points (3 replicates) and cross-linked under vacuum in 25 ml ice-cold MC buffer (1% (v/v) formaldehyde) for 2 × 10 min. For chromatin extraction, frozen tissue was ground, resuspended in 25 ml M1 buffer, and filtered through Miracloth mesh (Merck), washed five times in 5 ml M2 buffer, and once in 5 ml M3 buffer with a 10 min, 4 °C, 1000 g centrifugation step in between washing steps. The chromatin pellet was resuspended in 1 ml sonication buffer and sonified using a Diagenode Bioruptor (17 cycles of 30 s on/off at low intensity settings). Chromatin was incubated with anti-DYKDDDDK paramagnetic beads (Miltenyi Biotec) for 1.5 h at 4 °C and recovered with DYKDDDDK isolation kit (Miltenyi Biotec). For ChIP-qPCR, immunoprecipitated DNA was quantified as described [22].

### Library preparation and quantification

DNA libraries for each sample were prepared from isolated chromatin using the NEBNext Ultra II DNA Library Prep Kit for Illumina (NEB) and NEBNext Multiplex Oligos for Illumina (NEB) according to the manufacturer’s instructions. No size selection was performed after adaptor ligation. DNA was amplified by PCR with 11 amplification cycles. Library concentration and fragment size distribution were checked using D1000 ScreenTape with TapeStation bioanalyzer (Agilent). Library quantitation was done with the NEBNext Library Quant Kit for Illumina (NEB) according to the manufacturer’s instructions. Pooled libraries were sequenced on an Illumina NextSeq 500 with 75 bp SE reads.

### DAP-seq

Genomic DNA was isolated from Col-0 seedlings using CTAB method, digested with RNase A, and cleaned using phenol to chloroform to isoamyl alcohol extraction. Isolated genomic DNA was sonicated to an average length of 200 bp in Covaris sonicator and quantified. After sonication, fragmented genomic DNA was end prepared and adaptor ligated according to the protocol of NEBNext Ultra II DNA Library Prep Kit for Illumina (E7645) and purified using NaOAc and ethanol. The DNA was diluted in Elution buffer (10 mM Tris–HCl, pH 8.5) and measured using Qubit dsDNA HS Assay Kit; 200–300 ng prepared DNA was used for each binding reaction.cDNA sequences of *HSFA1b*, *HSFA2*, and *HSFA3* were subcloned into *pIX-HALO*. Oligonucleotide sequences are available in Additional File 1: Table S1. HALO-tagged HSFs were expressed in the TNT wheat germ expression system (Promega). Different combinations of HSFs or HALO (negative control) were incubated with prepared DNA overnight at room temperature. For 37 °C treatment, the samples were treated at 37 °C for 45 min before overnight incubation at room temperature. Washing and DNA recovery steps were as described [54]. The recovered DNA was amplified using Q5 Master Mix and different index primers in NEBNext® Ultra™ II DNA Library Prep Kit, pooled and size selected (200–700 bp) by gel fractionation; 10 nM DNA library was sequenced using Illumina NextSeq 500 with 150 bp SE reads.

### Bioinformatics analysis

If not mentioned otherwise, data analyses were done using R (https://www.r-project.org/) version 3.52. Data visualizations were done using the R lattice (version 0.20.41) and latticeExtra (version 0.6.28) packages (http://lmdvr.r-forge.r-project.org/). Enrichments within clusters were tested using a Fisher’s exact test with 2 × 2 contingency tables, R fisher.test function with alternative = ”greater”. Distribution shifts visualized in empirical cumulative distribution function (ECDF) or density plots were tested by pairwise KS-tests, R ks.test function with alternative = ”less”. Results are given in row name distribution compared to column name distribution.

### ChIP-seq mapping, visualization, peak calling, quantification, gene association, and clustering

ChIP-seq reads were mapped against the *A. thaliana* reference genome (TAIR10) using bwa mem [55] (http://arxiv.org/abs/1303.3997) version 0.7.12-r1044. Mappings were sorted and indexed using samtools version 1.3.1 [56]. Mapping files of biological replicates were merged using samtools version 1.3.1 to generate normalized coverage tracks using deepTools [57] version 1.50 bamCoverage function with the following parameters set: –binSize 10 –normalizeUsing RPGC –effectiveGenomeSize 120,654,995 –ignoreForNormalization chloroplast mitochondria –extendReads 75. Visualizations were done using IGV version 2.10.3 [58]. Peak calling was done for each sample using MACS [59] version 2.1.2. Peaks shorter than 500 bp were discarded. Peak summits ± 100 bp were extracted from all samples and merged using bedtools [60] version 2.29.1 merge, giving peak summit regions we call peaks. Reads mapping to peaks were counted for all samples using bedtools multicov. Samples 3-A3-NTC_S9 and 2-A3-4h_S10 were identified as outliers based on hierarchical clustering using the R hclust function and excluded from follow-up analyses. Peaks with more than 10,000 reads over all samples were excluded from follow-up analyses. Remaining read counts were normalized to counts per million (CPM). Distances to the closest transcription start site (TSS) were calculated using bedtools closest. Genes less than 2000 bp away were associated to the corresponding peak. Peak log2(CPM + 1) values were clustered using the R hclust function. Grouping in 15 clusters was selected.

### Motif analysis

Motifs were identified within peak summit regions of ChIP-seq clusters 7, 11, 12, and 14 using homer [61] version 4.11 findMotifsGenome.pl with the following parameters: -size given -len 13,15,17,6,8,10,12 -mis 4 -S 10. Fractions of peaks with de novo and the top 23 known motifs were quantified within each cluster. Motif enrichments within 500 bp upstream of the start of Col-0 memory genes (1–1-1) overlapping with ChIP-seq clusters 7, 12, and 14 and RNA-seq clusters 11, 14, and 15 compared to all other Col-0 memory genes were calculated giving the corresponding coordinate files, -bg for the second one.

### DAP-seq mapping and quantification

DAP-seq reads were mapped against the *A. thaliana* reference genome (TAIR10) using bwa mem [55] version 0.7.12-r1044. Mappings were sorted and indexed using samtools [56] version 1.3.1. Reads mapping against ChIP-seq peak summit regions were counted using bedtools [60] version 2.29.1 multicov and CPM-normalized.

### RNA-seq analysis, clustering

We re-analyzed our data published in [22]. We did analogous analyses to those described in [22], yet also including transposable element genes. Reads were mapped against the *A. thaliana* reference genome (TAIR10) using STAR [62] version 2.5.1a using the –quantMode GeneCounts opition to get gene counts. Differential gene expression analysis was done using DESeq2 [63] version 1.22.2. Col-0 memory genes (1–1-1) were defined as being significantly up-regulated at 4 h, 28 h, and 52 h after acclimation treatment (log2(fold change) > 1, *p* < 0.05). Col-0 partial memory genes (1–1-0) were defined as being significantly up-regulated at 4 h and 28 h but not at 52 h after acclimation treatment. Col-0 1–0-0 genes were defined as being significantly up-regulated at 4 h but not at 28 h and 52 h after acclimation treatment. Read counts were tags per million (TPM) normalized taking into account the length of the major annotated transcript form (TAIR10). log2(TPM + 1) values were clustered using the R hclust function. Grouping in 15 clusters was selected.

### Chromatin states and histone modifications

Positional chromatin states as defined by [43] were overlapped with ChIP-seq peak summit regions using bedtools version 2.29.1 intersect. The proportion of peaks within each cluster overlapping with regions associated to the different states was calculated. H3, H3K27me3, H3K36m3, H3K4me3, H3K9me2, and H3PanAc histone modification and input data from [42] was downloaded from NCBI GEO, accession number GSE143835, in bigWig format. Median coverage profiles around peak summits ± 1500 bp were computed for all 15 ChIP-seq cluster using deepTools [57] version 3.5.0 computeMatrix with the following parameters: reference-point –referencePoint center -b 1500 -a 1500. Raw fastq files for H3K4me3 and H3K27me3 histone modification data with and without heat acclimation from [47] were downloaded from NCBI SRA, project accession number PRJDB11556. Reads were mapped against the *A. thaliana* reference genome (TAIR10) using bwa mem version 0.7.12-r1044. Mappings were sorted and indexed using samtools version 1.3.1. Normalized coverage tracks were made using deepTools as for the ChIP-seq data but with the extendReads parameter set to 126. Median coverage profiles along selected groups of genes ± 500 bp were calculated for all histone modification data using the deepTools computeMatrix program with the following parameters: scale-regions –regionBodyLength 2000 -b 500 -a 500. Reads mapping against TSS + 700 bp regions were counted for each sample using bedtools intersect and CPM normalized. RNA-seq log2(TPM + 1) in Col-0 NHS samples were plotted by log2(CPM + 1) of those counts for each RNA-seq cluster.

## Supplementary Information

**Additional file 1: Fig. S1.** *pHSFA2::3xFlag-HSFA2* complements the *hsfa2* mutant phenotype. **Fig. S2.** Read profiles of representative peaks from selected ChIP-seq clusters. **Fig. S3.** Independent validation of representative peaks by ChIP-qPCR. **Table S1.** Oligonucleotides used in this study.

**Additional file 2: Supplementary Data 1.** Statistical analyses.

**Additional file 3: Supplementary Data 2.** ChIP-seq clustering genes.

**Additional file 4: Supplementary Data 3.** *Cis*-element motif analysis.

**Additional file 5: Supplementary Data 4.** RNA-seq clustering genes.

**Additional file 6: Supplementary Data 5.** Venn overlap gene list.

**Additional file 7. **Peer review history.

## Data Availability

ChIP-seq and DAP-seq data have been deposited at NCBI GEO under accession numbers GSE192427 [64] and GSE192407 [65], respectively. RNA sequencing data have been deposited under accession number GSE162434 [66]. The plant materials generated during this study are available upon request.
