# Supplementary material for: Genomic and epigenomic determinants of heat stress-induced transcriptional memory in Arabidopsis
Source: Genome Biol. 2023 May 30;24:129. doi: 10.1186/s13059-023-02970-5 (PMC10230730; doi:10.1186/s13059-023-02970-5)
Supplement: Supplementary file 1 — Additional file 1: Fig. S1. pHSFA2::3xFlag-HSFA2 complements the hsfa2 mutant phenotype. Fig. S2. Read profiles of representative peaks from selected ChIP-seq clusters. Fig. S3. Independent validation of representative peaks by ChIP-qPCR. Table S1. Oligonucleotides used in this study. [file 13059_2023_2970_MOESM1_ESM.pdf]

Kappel et al., Additional File 1: Fig. S1

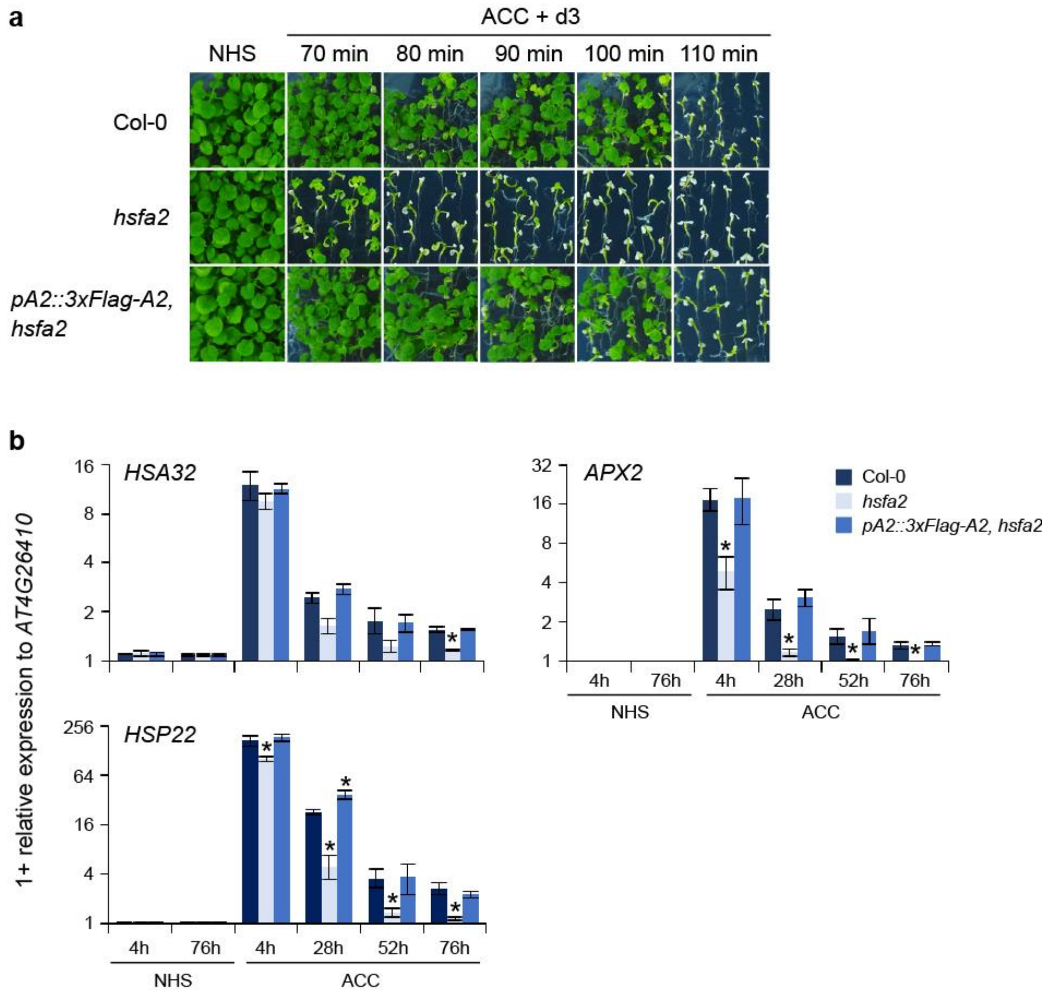

Fig. S1: *pHSFA2::3xFlag-HSFA2* complements the *hsfa2* mutant phenotype.

(**a**) HS memory assay. 4 d-old seedlings of the indicated genotypes were either subjected to a 2-step acclimation (ACC) treatment (cf. Fig. 1a) which was followed 3 days later by exposure to 44°C HS for 70-110 min, or no HS (NHS). Pictures were taken 14 days after ACC and are representative of three independent experiments. (**b**) Transcript levels of HS memory genes *HSA32*, *HSP22.0* and *APX2* as measured by qRT-PCR. 4 d-old seedlings were either not treated (NHS) or subjected to ACC treatment and sampled at the indicated time points. Expression values were normalized to the *At4g26410* reference. Data are mean ± SD of three independent experiments. Asterisks indicate statistically significant differences to Col-0 (*, $p < 0.01$; unpaired two-sided t-test).

Kappel et al., Additional File 1: Fig. S2

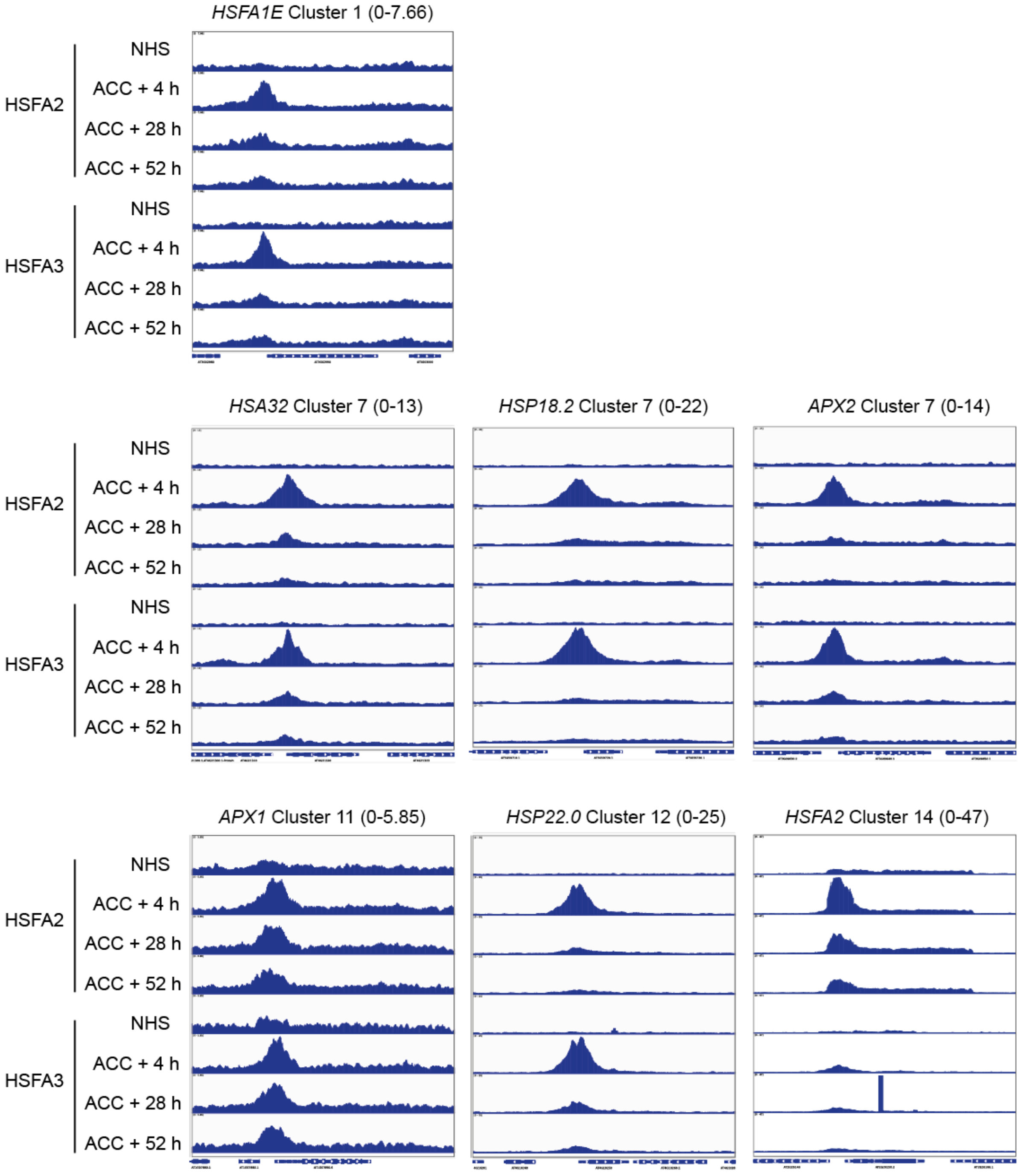

Fig. S2: Read profiles of representative peaks from selected ChIP-seq clusters.

Normalized cumulative read profiles for representative genes from selected ChIP-seq clusters c1 (*HSFA1E*), c7 (*HSA32*, *HSP18.2*, *APX2*), c11 (*APX1*), c12 (*HSP22.0*), c14 (*HSFA2*) were generated and aligned to the genome. The indicated gene is the one displayed in the center of the panel. The y-axis scale is maintained constant for each gene and the range is indicated in the title of each panel.

Kappel et al., Additional File 1: Fig. S3

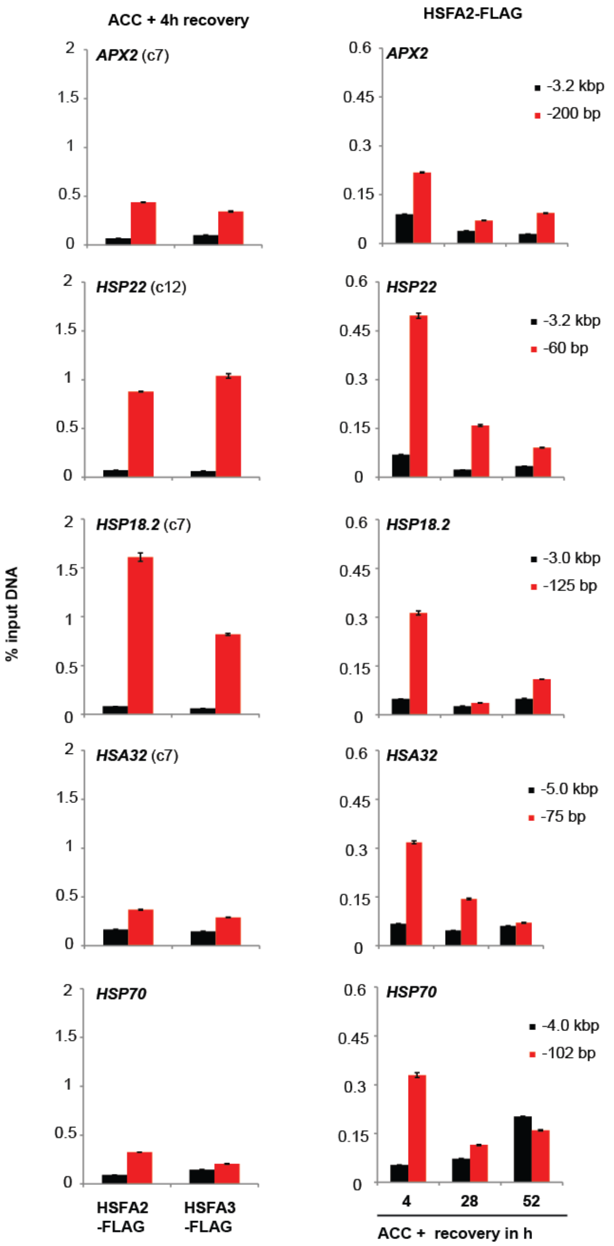

Fig. S3: Independent validation of representative peaks by ChIP-qPCR.

(left) Occupancy of HSFA2 and HSFA3 at selected memory genes as determined by ChIP-qPCR of *pHSFA2::FLAG-HSFA2* and *pHSFA3::FLAG-HSFA3* lines. Samples were crosslinked at ACC + 4 h recovery. (right) Occupancy of HSFA2 at selected memory genes at the indicated time points of the *pHSFA2::FLAG-HSFA2* line. Samples were crosslinked at ACC + 4, 28 or 52 h recovery. Enrichment was normalized to input (data are mean ± SD of three replicates). For each gene one positive (red) and one negative control amplicon (black) are shown.

Kappel et al., Additional File 1: Table S1: Oligonucleotide sequences

2624/pHSFA2-AscI-F	AGGCGCGCCCTGTTTGGTTATCGGGTGAG AGAAAAATTG	*HSFA2* complementation line
2625/pHSFA2-AgeI-R	TACCGGTTTTCGTTGTTTATCTCAAATCCAT AAGCTCAG	*HSFA2* complementation line
3268/AgeI-3xFLAG-gHSFA2-NotI_	AACCGGTATGGACTACAAAGACCATGACG GTGATTATAAAGATCATGATATCGATTACAA GGATGACGATGACAAGGGAGCAGGAGCAA TGGAAGAACTGAAAGTGGAAATGG	*HSFA2* complementation line
3269/AgeI-3xFLAG-gHSFA2-NotI_R	TGCGGCCGCATTTCTCTTTCTTATCCTTAAA ATCCC	*HSFA2* complementation line
1547/F-AT4G26410	GAGCTGAAGTGGCTTCAATGAC	qRT-PCR
1548/R-AT4G26410	GGTCCGACATACCCATGATCC	qRT-PCR
352/LP-Hsa32-leftIntron4	CTGATGCGAAGTTGGTTGAG	qRT-PCR
353/RP_Hsa32-3UTR	GCACATAACATCAGACACATACGA	qRT-PCR
633/AT3G09640 REV	ACTCCTTGTCAGCAAACCCGAG	qRT-PCR
634/AT3G09640 FOR	CTTGATGATCCTCTCTTTCTCCCA	qRT-PCR
637/AT4G10250 REV	TTCAGGAGATAGTTTCGTGAGGTTA	qRT-PCR
638/AT4G10250 FOR	ATTCTGGAGACAGTTCAAGCTACCT	qRT-PCR
267/HSP101F	ATGACCCGGTGTATGGTGCTAG	qRT-PCR
268/HSP101R	CGCCTGCATCTATGTAAACAGTG	qRT-PCR
4116 / HSFA1b-_attB1_F1	GGGGACAAGTTTGTACAAAAAAGCAGGCT TAATGGAATCGGTTCCCGAATC	Gateway cloning of HSFA1b cDNA into pIX-HALO
4117 / HSFA1b_attB2_R1	GGGGACCACTTTGTACAAGAAAGCTGGGT TTTATTTCCTCTGTGCTTCTG	
4120 / HSFA2_attB1_F1	GGGGACAAGTTTGTACAAAAAAGCAGGCT TAATGGAAGAACTGAAAGTG	Gateway cloning of HSFA2 cDNA into pIX-HALO
4121 / HSFA2_attB2_R1	GGGGACCACTTTGTACAAGAAAGCTGGGT TTTAAGGTTCCGAACCAAG	
4122 / HSFA3_attB1_F1	GGGGACAAGTTTGTACAAAAAAGCAGGCT TAATGAGCCCAAAAAAAGATG	Gateway cloning of HSFA3 cDNA into pIX-HALO
4123 / HSFA3_attB2_R1	GGGGACCACTTTGTACAAGAAAGCTGGGT TCTAAGGATCATTCATTGG	
