# Supplementary material for: Genomic and epigenomic determinants of heat stress-induced transcriptional memory in Arabidopsis
Source: Genome Biol. 2023 May 30;24:129. doi: 10.1186/s13059-023-02970-5 (PMC10230730; doi:10.1186/s13059-023-02970-5)
Supplement: Supplementary file 7 — Additional file 7. Peer review history. [file 13059_2023_2970_MOESM7_ESM.docx]

**Review history**

**First round of review**

**Reviewer 1**

This work presents a correlation between HSF-dependent transcription and chromatin in the context of heat stress memory. Although the authors have conducted a detailed analysis of the data, the work lacks deep statistical analysis and/or experimental validation.
Major concerns:
- The authors describe the potential targets of HSFA2 and HSFA3 but this analysis is descriptive and should include experimental validation.
- The correlation between transcription and chromatin profiles is limited to a very small number of gene, and does not include a statistical analysis nor experimental validation.
- It is currently unclear how the DAP-seq analysis helps to determine the binding strength of the transcription factors tested; a ChIP analysis under different conditions or mutant analysis would have been more appropriate.
- The integration of expression and chromatin data is largely descriptive, thus it is difficult to distinguish between cause and effect.

**Reviewer 2**

Heat stress (HS) induces transcriptional memory that allows plants to respond more efficiently to a recurrent HS. At the molecular level, priming against HS is associated with two types of transcriptional memory: (1) the sustained induction of HS-induced gene expression that lasts several days after the priming HS (type I), and (2) enhanced transcriptional re-induction after a recurrent HS (type II). Genes that show type I and type II transcriptional memory after HS have been catalogued and several factors have been identified that are necessary for the transcriptional memory. However, the underlying molecular mechanisms are not clear, as is the question what distinguishes memory genes from HS-inducible non-memory genes.

In this work, Kappel et al. try to address this issue. For this, the authors identified global target genes of two key memory heat shock transcription factors, HSFA2 and HSFA3, using timecourse ChIP-seq. They identified a large number of peaks, suggesting that not all may be functionally relevant. To select relevant targets they combined different analysis.

1) They performed hierarchical clustering to sort the peaks in different categories according to the binding profile over the time course. They obtained 15 clusters (cclusters). According to their median signal strength, they found 3 groups: 1-(c1-5 and c8) have low signal, 2-(c7,c9-12,c15) intermediate signal and 3-(c6,c13,c14) strong signal. Since HSFA2 and HSFA3 expression is HS inducible, true targets may show low (or no) binding in NHS conditions. c1, c2, c5, c7, c11, c12, c14 showed an increase in signal strength after HS, these clusters comprised 4948 peaks, which is still a very large number. A large fraction of peaks was found close to a TSS and another fraction several hundred to more than 1,000 bp away. c7 and c12 contained much higher proportion of peaks at the TSS.

2) They analyzed the average profile of 5 frequently profiled histone modifications
148 (H3PanAc (acetylation), H3K4me3, H3K9me2, H3K27me3, H3K36me3). All clusters with a HS induced increase in HSF binding (clusters c1, c2, c5, c7, c11, c12, c14) are enriched in open chromatin that is typical for promoter regions.

3) They asked whether the increased binding of memory HSFs to genes in clusters c1, c2, c5, c7, c11, c12, c14 is reflected in induced gene expression after HS. Thus, they integrated the ChIP-seq data with transcript profiling data of Col-0, hsfa2, hsfa3 and hsfa2, hsfa3 double mutants at the corresponding time points (NHS, 4 h, 28 h, 52 h after ACC). Most probable target genes are those which expression is upregulated at 4 h after ACC, their induction is sustained at 28 and 52h and that this is dependent on HSFA2/HSFA3. They found that c12 and c14 and to a lesser extent c7 are enriched for these genes. c12 has 43 genes, c14 contain HSFA2 and its neighbor gene (2 genes), and c7 contain 310 genes, which include several previously characterized HS-memory genes, such as APX2, HSA32, and HSP18.2.

4) They performed DAP-seq and Motif analysis of sequences underlying ChIP-seq peaks. Despite no single motif fully discriminated between peaks in memory vs. non-memory clusters, they showed that strong binding of HSFA2 and HSFA3 to the tripartite HSE motif TTCtaGAAnnTTCt contributes to the sustained expression of heat-stress memory

5) They performed and orthogonal clustering analysis based on transcriptome datasets covering the same treatments and time points, and all mutant combinations. Since HSFA2 and HSFA3 are transcriptional activators that regulate HS memory by maintaining sustained gene expression, the expression of direct target genes is expected to be sustained after ACC and less sustained and possibly less induced in hsfa2 and hsfa3 mutants. Of the 15 RNA-seq clusters (rclusters), three were enriched in strongly HS-inducible genes with sustained expression; all three were dependent on both HSFA2 and HSFA3: rclusters r11 (n=45), r14 (n=14), r15 (n=1).

6) They compared previously defined HS memory genes showing upregulation at 4 h after ACC and sustained induction that lasts until 52 h into the recovery phase (1-1-1), with memory gene sets defined by expression cluster analysis and memory gene sets based on HSFA2/HSFA3 binding, finding strong overlap.


In summary, using a very elegant combination of analyses, the authors identify memory targets of HSFA2/A3. Although they were unable to identify a strictly memory-specific HSE motif, they show that these genes are characterized by a combination of six features. The analyses are clearly explained and performed. In my opinion, this or similar combination of analyses should be required to identify transcription factors functionally relevant targets in general. I do not have major concerns on the manuscript, only few suggestions:

I would suggest including Venn diagrams showing overlap among c12_c14_c7 group of genes, r11_r14_ r15 group of genes and 1-1-1, 1-1-0 or 1-0-0 genes.

Did the authors checked whether the overlapping genes contain any specific DNA motifs or any other feature that differentiate them from non-memory genes?

Line 186 - remove {Citation}

**Authors’ response to reviewers**

Reviewer #1: This work presents a correlation between HSF-dependent transcription and chromatin in the context of heat stress memory. Although the authors have conducted a detailed analysis of the data, the work lacks deep statistical analysis and/or experimental validation.

> We have added statistical analysis to the findings presented in Figs. 1c, 2e, 3a, 3b, 6b, 7a, 7c, 7f, 8b, 8d, 8f (summarized in Supplementary Data 1) and experimental validation (Supplementary Figure S3) as detailed below.

Major concerns:
- The authors describe the potential targets of HSFA2 and HSFA3 but this analysis is descriptive and should include experimental validation.

> We added experimental validation for five target genes from the overlap between the ChIP –seq, RNA-seq clusters and transcriptomic HS-responsive genes as Supplementary Figure S3 (l. 196-199). In addition, our findings are fully consistent with previously published ChIP-qPCR data with HSFA2- and/or HSFA3-tagged transgenic lines on HSP21, HSP22.0, HSP70, HSP18.2, HSA32 (Lämke et al., 2016; Friedrich et al., 2021). Thus, the ChIP-seq analysis presented here is fully supported by independent ChIP-qPCR analysis.

- The correlation between transcription and chromatin profiles is limited to a very small number of gene, and does not include a statistical analysis nor experimental validation.

> In Fig. 2e we analyze the enrichment of chromatin states at the ChIP-seq peaks in a cluster-wide analysis. We conclude from this analysis is that some ChIP-seq clusters are slightly enriched in open chromatin states 1 and 2 (l.166-168). We have added statistical analysis to Fig. 2e. For all cluster-based analyses, the number of elements in the cluster (Peaks or genes) determines the number of genes.
In Fig. 8 we analyzed the enrichment of H3K4me3 after HS and long recovery (3 days) and found enrichment for several cclusters/rclusters/groups. These were statistically significant as determined by KS-tests (Fig. 8b, d, f and corresponding legends). We have clarified the statistical analysis in the main text (l.310-314, 319-321), in addition to the figure legend. The results of the statistical analysis are detailed in Supplementary Data 1.
APX2, HSP22, HSP18.2 and HSP21 are genes in the intersection of HSFA2/A3 binding, HSFA2/3-dependent expression and sustained induction (“1-1-1”). The sustained enrichment of H3K4me3 at these genes was previously demonstrated by ChIP-qPCR (Lämke et al., 2016; Friedrich et al., 2021, Liu et al., 2018) and is fully consistent with the present analysis (l. 304-305).

- It is currently unclear how the DAP-seq analysis helps to determine the binding strength of the transcription factors tested; a ChIP analysis under different conditions or mutant analysis would have been more appropriate.

> DAP-seq provides a complementary way to assess DNA binding of transcription factors. The primary goal of our DAP-seq analysis was to assess the contribution of chromatin structure and complex composition to the binding of HSFA2 and HSFA3 to their genomic targets. DAP-seq is well suited for this purpose as it determines the genome-wide binding of a single - or a combination of - transcription factor(s) to chromatin-free DNA. The overall high similarity of binding patterns indicates that DNA sequence is the major determinant of binding. The lack of additional transcription factors in the DAP-seq binding reaction ruled out major influences of additional unidentified factors for the binding of HSFA2 and HSFA3. The overall binding intensity per cluster was strikingly similar in DAP-seq compared to ChIP-seq, indicating that DNA sequence is the primary determinant of binding intensity. The significance of the analysis is summarized in l. 215-220 and discussed in l. 368-383.


- The integration of expression and chromatin data is largely descriptive, thus it is difficult to distinguish between cause and effect.

> Our data provide a clear correlation of HS-induced H3K4me3 enrichment and memory gene behavior at the genomic level. In addition, we show that memory genes are lowly expressed before HS and show an exquisite correlation with pre-HS H3K4me3 levels (Fig. 9). Decades of research have failed to disentangle the question whether transcription drives chromatin dynamics or vice versa. Due to the intricate connections, perturbation of one usually also perturbs the other without clearly establishing a hierarchy of effect. Previous works indicated that loss of transcription factors (and hence expression) also causes loss of chromatin modifications. For example, it was previously shown that the hsfa2 hsfa3 double mutant fails to accumulate sustained H3K4me3 at HSP22 and APX2 (Friedrich et al., 2021). The current study adds a genome-wide perspective to this. It also clearly indicates that transcription is not sufficient for high H3K4me3 enrichment, otherwise this would also be present in 1-0-0 genes.


Reviewer #2: Heat stress (HS) induces transcriptional memory that allows plants to respond more efficiently to a recurrent HS. At the molecular level, priming against HS is associated with two types of transcriptional memory: (1) the sustained induction of HS-induced gene expression that lasts several days after the priming HS (type I), and (2) enhanced transcriptional re-induction after a recurrent HS (type II). Genes that show type I and type II transcriptional memory after HS have been catalogued and several factors have been identified that are necessary for the transcriptional memory. However, the underlying molecular mechanisms are not clear, as is the question what distinguishes memory genes from HS-inducible non-memory genes.

In this work, Kappel et al. try to address this issue. For this, the authors identified global target genes of two key memory heat shock transcription factors, HSFA2 and HSFA3, using timecourse ChIP-seq. They identified a large number of peaks, suggesting that not all may be functionally relevant. To select relevant targets they combined different analysis.

1) They performed hierarchical clustering to sort the peaks in different categories according to the binding profile over the time course. They obtained 15 clusters (cclusters). According to their median signal strength, they found 3 groups: 1-(c1-5 and c8) have low signal, 2-(c7,c9-12,c15) intermediate signal and 3-(c6,c13,c14) strong signal. Since HSFA2 and HSFA3 expression is HS inducible, true targets may show low (or no) binding in NHS conditions. c1, c2, c5, c7, c11, c12, c14 showed an increase in signal strength after HS, these clusters comprised 4948 peaks, which is still a very large number. A large fraction of peaks was found close to a TSS and another fraction several hundred to more than 1,000 bp away. c7 and c12 contained much higher proportion of peaks at the TSS.

2) They analyzed the average profile of 5 frequently profiled histone modifications
148 (H3PanAc (acetylation), H3K4me3, H3K9me2, H3K27me3, H3K36me3). All clusters with a HS induced increase in HSF binding (clusters c1, c2, c5, c7, c11, c12, c14) are enriched in open chromatin that is typical for promoter regions.

3) They asked whether the increased binding of memory HSFs to genes in clusters c1, c2, c5, c7, c11, c12, c14 is reflected in induced gene expression after HS. Thus, they integrated the ChIP-seq data with transcript profiling data of Col-0, hsfa2, hsfa3 and hsfa2, hsfa3 double mutants at the corresponding time points (NHS, 4 h, 28 h, 52 h after ACC). Most probable target genes are those which expression is upregulated at 4 h after ACC, their induction is sustained at 28 and 52h and that this is dependent on HSFA2/HSFA3. They found that c12 and c14 and to a lesser extent c7 are enriched for these genes. c12 has 43 genes, c14 contain HSFA2 and its neighbor gene (2 genes), and c7 contain 310 genes, which include several previously characterized HS-memory genes, such as APX2, HSA32, and HSP18.2.

4) They performed DAP-seq and Motif analysis of sequences underlying ChIP-seq peaks. Despite no single motif fully discriminated between peaks in memory vs. non-memory clusters, they showed that strong binding of HSFA2 and HSFA3 to the tripartite HSE motif TTCtaGAAnnTTCt contributes to the sustained expression of heat-stress memory

5) They performed and orthogonal clustering analysis based on transcriptome datasets covering the same treatments and time points, and all mutant combinations. Since HSFA2 and HSFA3 are transcriptional activators that regulate HS memory by maintaining sustained gene expression, the expression of direct target genes is expected to be sustained after ACC and less sustained and possibly less induced in hsfa2 and hsfa3 mutants. Of the 15 RNA-seq clusters (rclusters), three were enriched in strongly HS-inducible genes with sustained expression; all three were dependent on both HSFA2 and HSFA3: rclusters r11 (n=45), r14 (n=14), r15 (n=1).

6) They compared previously defined HS memory genes showing upregulation at 4 h after ACC and sustained induction that lasts until 52 h into the recovery phase (1-1-1), with memory gene sets defined by expression cluster analysis and memory gene sets based on HSFA2/HSFA3 binding, finding strong overlap.

In summary, using a very elegant combination of analyses, the authors identify memory targets of HSFA2/A3. Although they were unable to identify a strictly memory-specific HSE motif, they show that these genes are characterized by a combination of six features. The analyses are clearly explained and performed. In my opinion, this or similar combination of analyses should be required to identify transcription factors functionally relevant targets in general. I do not have major concerns on the manuscript, only few suggestions:

I would suggest including Venn diagrams showing overlap among c12_c14_c7 group of genes, r11_r14_ r15 group of genes and 1-1-1, 1-1-0 or 1-0-0 genes.

> We have added the suggested Venn diagrams to Fig. 7h and Supplementary Data 5. The overlap between individual c and r clusters is displayed in Fig. 6b.

Did the authors checked whether the overlapping genes contain any specific DNA motifs or any other feature that differentiate them from non-memory genes?

> We show the motif analysis (known motifs and de novo motifs) of the ChIP-seq clusters in Fig. 5. Based on the reviewer’s suggestion we reanalyzed whether any motifs are enriched in the genes that are found in the overlap between (c7, c12, c15), (r11, r14, r15) and 1-1-1 and are thus directly bound, HSFA2/A3-dependent in their transcription and induced for at least two days after HS. Compared to the rest of 1-1-1 genes the overlapping genes are even more enriched for HSEs (Supplementary Data 5). However, we were not able to further identify any subspecialization of the HSE sequences.

Line 186 - remove {Citation}
> removed.

**Second round of review**

**Reviewer 2**

The authors have been addressed my points in this new version of the manuscript.
